# Evaluation of DNA damage induced by ionizing radiation from myocardial perfusion imaging: a pilot study

**DOI:** 10.1186/s12872-022-02839-8

**Published:** 2022-09-03

**Authors:** Anna Paula Arpini, Andrea De Lorenzo, Aniele Moritz, Julia Passarelli Pereira, Glauber Monteiro Dias

**Affiliations:** 1 Coordenação de Ensino E Pesquisa, Instituto Nacional de Cardiologia, Rua das Laranjeiras 374, Rio de Janeiro, RJ Brazil; 2 Laboratório de Biologia Celular E Tecidual, Centro de Biociências E Biotecnologia, Universidade Estadual Do Norte Fluminense Darcy Ribeiro, Rio de Janeiro, RJ Brazil

**Keywords:** Myocardial perfusion imaging, Radiation, Comet assay, DNA

## Abstract

**Background:**

As patient exposure to ionizing radiation raises concern about malignancy risks, this study evaluated the effect of ionizing radiation on patients undergoing myocardial perfusion imaging (MPI) using the comet assay, a method for detection of DNA damage.

**Methods:**

Patients without cancer, acute or autoimmune diseases, recent surgery or trauma, were studied. Gated single-photon myocardial perfusion imaging was performed with Tc-99m sestamibi. Peripheral blood was collected before radiotracer injection at rest and 60–90 min after injection. Single-cell gel electrophoresis (comet assay) was performed with blood lymphocytes to detect strand breaks, which determine a “comet tail” of variable size, visually scored by 3 observers in a fluorescence microscope after staining (0: no damage, no tail; 1: small damage; 2: large damage; 3: full damage). A damage index was calculated as a weighted average of the cell scores.

**Results:**

Among the 29 individuals included in the analysis, age was 65.3 ± 9.9 years and 18 (62.1%) were male. The injected radiotracer dose was 880.6 ± 229.4 MBq. Most cells (approximately 70%) remained without DNA fragmentation (class 0) after tracer injection. There were nonsignificant increases of classes 1 and 2 of damage. Class 3 was the least frequent both before and after radiotracer injection, but displayed a significant, 44% increase after injection.

**Conclusion:**

While lymphocytes mostly remained in class 0, an increase in class 3 DNA damage was detected. This may suggest that, despite a probable lack of biologically relevant DNA damage, there is still a need for tracer dose reductions in MPI.

## Background

Medical imaging- and, particularly, cardiac imaging- grew steadily from late 1980s to early 2000s, raising concern about patients´ exposure to ionizing radiation and carcinogenesis [1–3]. The 2019 National Council on Radiation Protection and Measurements (NCRP) Report, which updated medical radiation exposure information with data collected between 2006 and 2016, showed that Nuclear Medicine still accounted for 15% of the radiation burden, even though a decrease was noted [4]. While current practice is generally aligned with patient-centered imaging and radiation safety, the effects of ionizing radiation from imaging studies still merit attention.

To understand the potentially harmful effects of ionizing radiation from medical imaging, several studies have, for a long time, tried to make correlations between radiation exposure in medical, occupational and accidental contexts and cytogenetic alterations. However, radiation doses vary largely among studies and are sometimes very large when compared to actual medical imaging [5–8]. More recent studies are unable to fill all the knowledge gaps in this field, either due to limited number of subjects or specific conditions (e.g., pediatric populations) [9, 10]. Additionally, many variables influence radiation sensitivity, including cell sensitivity to induction of DNA damage, differences in DNA repair, in cell growth and in proportions of cells in different phases of the cell cycle [11]. Therefore, there is an open field for the continuous evaluation of the effects of ionizing radiation on human DNA.

This study aimed to evaluate the occurrence of DNA damage in patients undergoing myocardial perfusion imaging (MPI) using the comet assay (alkaline single-cell gel electrophoresis). The assay is based on the lysis of the cell membrane, followed by the induction of electrophoretic migration in an agarose matrix [12], resulting in the transport of DNA fragments out of the nucleus. The image of DNA migration obtained resembles a comet with a head and a tail, hence the term comet assay [12, 13]. Using microscope evaluation, it is possible to observe and grade the proportion of DNA strands or fragments which migrated, classifying the degree of damage and turning this relatively easy and low-cost procedure an interesting option to detect DNA lesions in individual cells. Thus, the study may add data to the continuum of knowledge gained on that subject, with a focus on a largely employed diagnostic imaging method.

## Methods

Patients ≥ 18 years undergoing MPI at a single Nuclear Medicine laboratory were considered eligible for the study. Exclusion criteria were current or prior malignant neoplasm; autoimmune diseases; significant trauma, major surgery or exposure to radiation (diagnostic, therapeutic or occupational) in the past 3 months; acute infectious diseases or any acute disease with significant compromise of organs or systems (e.g. acute myocardial infarction, pulmonary embolism etc.).

The study was approved by the Ethics Committee of the Instituto Nacional de Cardiologia (# CAAE 6716971.6.0000.5272), and all patients provided written informed consent before participation in the study.

### Myocardial perfusion imaging

Gated single-photon emission computed tomography (SPECT) MPI was performed with Tc-99m sestamibi (8 MBq/kg) in a 2-day protocol. For this study, to avoid possible stress-induced effects on the DNA, patients were evaluated only in the rest phase of the MPI study, with the stress phase performed on a subsequent date to avoid any residual DNA damage from prior radiotracer injection. The first peripheral blood sample (4 ml) was collected before tracer injection, and the second, immediately before the patient left the Nuclear Cardiology laboratory after image acquisition (60–90 min after tracer injection). Images were acquired in a 2-head gamma camera (Infinia Hawkeye 4, General Electric Healthcare, WI, USA).

### Comet assay

The comet assay was performed according to the protocol described by Singh et al. [13]. Agarose-covered slides were prepared in duplicates (two with blood collected before, and two with blood collected after tracer injection). Each slide received a mixture of 5.0 µL of blood and 120.0 µL of low melting agarose, was covered with a coverslide, then refrigerated for five minutes for solidification, and thereafter the coverslides were removed. The slides were incubated in a lysis solution (1%Triton X-100, 10% dimethyl sulfoxide, 2.5 M NaCl, 100 mM ethylenediaminetetraacetic acid [EDTA], 10 mM Tris) for two hours, kept refrigerated and protected from light. Peripheral blood lymphocytes, which became nucleoid structures after lysis, were studied.

The alkaline unwinding, electrophoresis and neutralization steps were performed as described by Hartmann and Speit [14], with minor modifications. The slides were removed from the lysis solution and placed in the electrophoresis chamber, which was then filled with freshly made alkaline buffer (300 mM NaOH and 1 mM EDTA, pH 12.6). The cells were exposed to alkali for 40 min to allow for DNA unwinding and the expression of alkali-labile sites. Subsequently, the DNA was submitted to electrophoresis for 30 min at 300 mA and 25 V in an ice bath. All the above steps (preparation of slides, lysis and electrophoresis) were conducted without direct light in order to prevent additional DNA damage.

Positive controls were performed with 200 ml of whole human blood, incubated for 2 h at 37 °C with 50 ml of methyl methanesulfonate (final concentrations of 0.08 mM and 0.016 mM). The two concentrations were used to demonstrate different levels of damage and to ascertain the assay sensitivity.

After electrophoresis, the slides were placed in a horizontal position and washed three times (5 min each) with 0.4 M Tris buffer, pH 7.5, to neutralize the excess alkali. Finally, slides were fixed with absolute ethanol, stained with GelRed 1:500 (Biotium) and analyzed using a fluorescence microscope (Zeiss Axioplan with AxioCam MRc5 camera).

**Fig. 1:**
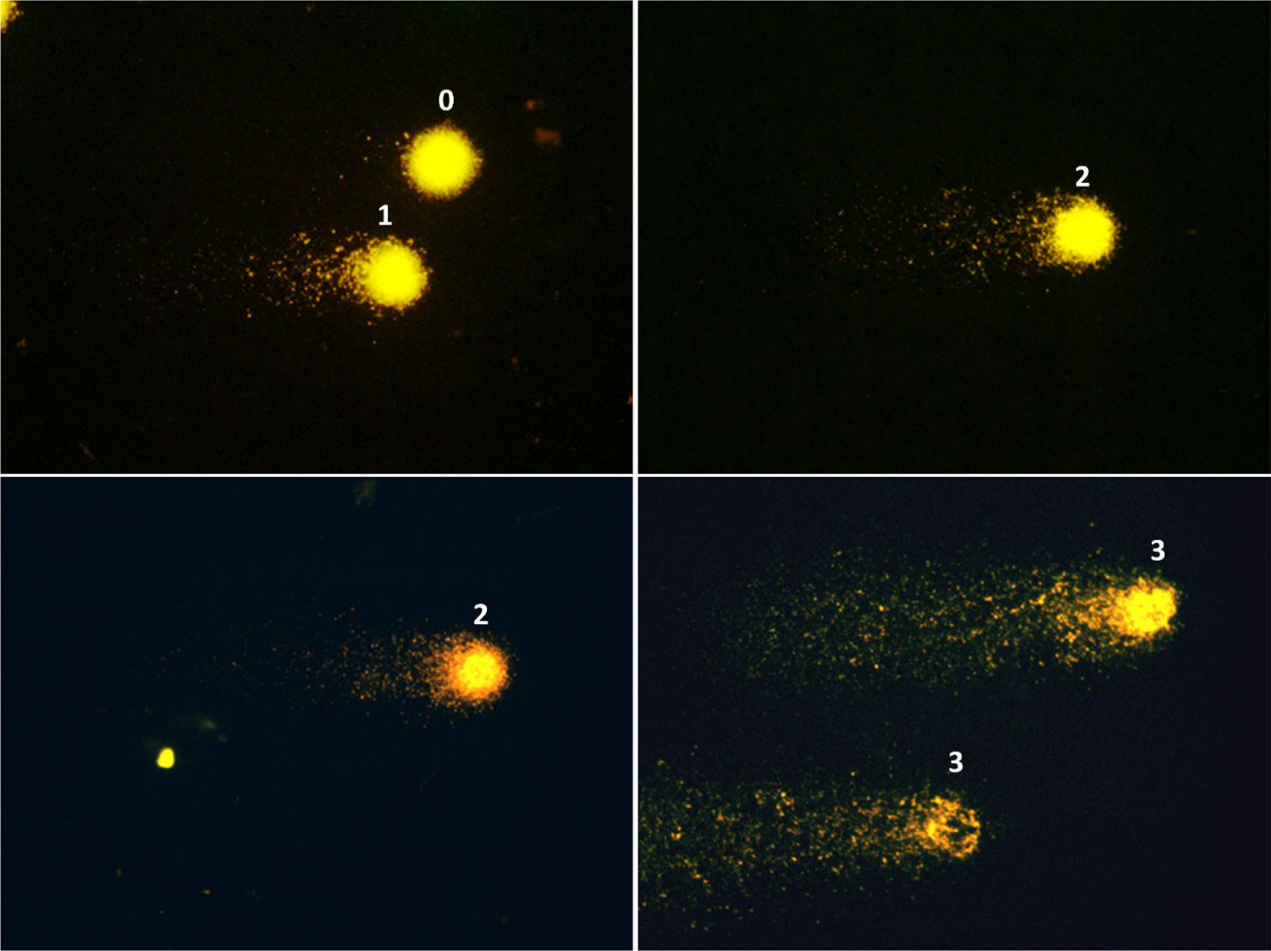
Fluorescence microscopy images (× 400) showing examples of patterns of DNA damage in the comet assay. Left panel: Nucleoid structures depicting class 0 of damage (no tail) and class 1 (small tail). Middle panel: class 2 of damage (larger tail). Right panel: class 3 of damage (profound fragmentation, very large tail)

The degree of DNA damage (strand breaks) was classified according to the size and intensity of the tails of the comets, into 4 classes: 0 (no damage- no tail), 1 (small damage, small tail), 2 (larger damage, large tail), and 3 (complete damage, with a small comet head and most of the DNA in the tail) **(**Fig. 1). The analysis was performed by 3 observers, who evaluated 100 cells in each slide; the mean of the scores from the 3 observers was used to calculate the damage index (DI), as shown below (where a = number of cells scored 0 by observer 1, and so on):

**Table:** 

Damage class	0	1	2	3
Observer 1	a	D	G	J
Observer 2	b	E	H	K
Observer 3	c	F	I	l
Final score	A (mean of a, b, c)	B (mean of d,e,f)	C (mean of g,h,i)	D (mean of j, k, l)

Damage index (DI) = (A × 0) + (B × 1) + (C × 2) + (D × 3)

The mean of the DI from the duplicate slides was used as the final value for each patient. Additionally, the frequency of the damage classes (0–3) was calculated from the total number of evaluated cells.

One hundred and twenty slides were evaluated, corresponding to the 30 patients with two slides from blood samples collected before tracer injection and two slides from the blood samples collected after tracer injection. One of the slides (sample 12) was removed from the final data analysis due to processing error.

### Statistical analysis

Variables with a normal distribution were demonstrated as mean and standard deviation; otherwise, they were demonstrated as medians interquartile ranges. Categorical variables were compared with chi-square, and continuous variables were compared with the Wilcoxon´s test. A value of *p* < 0.05 was considered statistically significant. Statistical analyses were performed using SPSS™ (version 22).

## Results

### Patient population

Among the 29 individuals included in the analysis, age was 65.3 ± 9.9 years (47–85 years), 18 (62.1%) were male, 28 (96.6%) were hypertensive, 13 (44.8%) were diabetic, 14 (48.3%) were dyslipidemic, 16 (55.2%) were former smokers, 7 (24.1%) had prior myocardial infarction, 13 (44.8%) had a history of myocardial revascularization (percutaneous or surgical), and 5 (17.2%) had stable, angiographically confirmed coronary artery disease undergoing medical treatment. The injected radiotracer dose was 880.6 ± 229.4 MBq.

### Comet assay

**Fig. 2:**
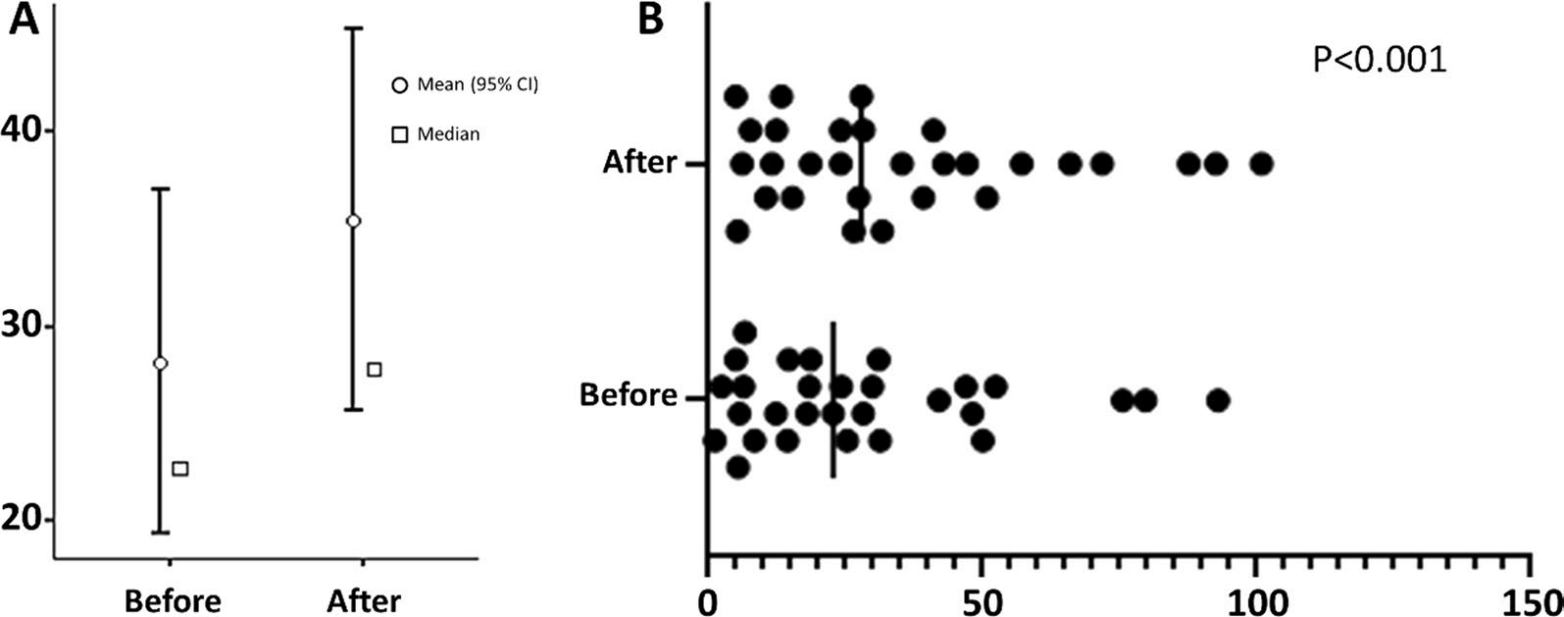
Damage index before and after radiotracer injection. **A** Means, medians and interquartile ranges. **B** Values of damage index from each patient

Overall, the medians of the DI before and after tracer injection were 22.7 and 27.8, respectively (*p* < 0.001) (Fig. 2 A). Figure 2B shows the DI of each patient.

Table 1 depicts the frequency of the damage classes before and after tracer injection. Most of the cells (approximately 70%) remained without DNA fragmentation (class 0) after tracer injection. There were nonsignificant increases of classes 1 and 2 of damage. Class 3 was the least frequent both before and after radiotracer injection, but displayed a significant, 44% increase after injection.

**Table 1:** Frequencies of damage classes before and after tracer injection

Damage class	Before (%)	After (%)	*p* value
0	76.45	70.27	0.4
1	20.01	25.06	0.4
2	2.69	3.45	0.06
3	0.85	1.23	0.046

## Discussion

Several types of stress, either environmental or organic, have genotoxic effects [15]. Ionizing radiation may promote oxidative stress, induce DNA strand breaks and affect cell components, even a few hours after irradiation [16, 17]. In medical imaging, radiation effects are considered stochastic, and mainly of two kinds, malignancy or heritable disease [18]. These effects are dose-dependent and follow a linear no-threshold model [19]. However, there is no direct evidence of cancer risk from cardiac imaging, but only projections from the epidemiological studies [20]. On the opposite end of this, studies at the “bench” level try to provide different types of evidence to help elucidate this issue.

The comet assay (single-cell gel electrophoresis) is one of the methods of choice for the evaluation and measurement of DNA damage. It is a simple, fast, precise, low-cost technique, in which cells are incorporated into an agarose matrix and then have their membranes lysed for the generation of nucleoid structures. Thereafter, DNA is untwined and undergoes electrophoresis. If there are bond breaks, the highly negative molecules move towards the anode [21, 22]. After staining and through visualization in a fluorescence microscope, a comet shape appears, with the nucleus in the head of the comet and the tail consisting of DNA strands or fragments which migrated to the anode. The relative intensity of the tail increases according to the intensity of damage caused by any agent, either ionizing radiation or chemical agents, for example [23].

In this study, even before exposure to ionizing radiation, 24% of the cells had evidence of some DNA damage, what recalls the variety of other factors that may lead to damage, such as smoking, diabetes, and indeed all currently recognized cardiovascular risk factors [24–26]. Importantly, even though there was an increase of the damage index and of classes 1–3 of damage, most cells remained in class 0. Shirazi et al. [9], also using the comet assay, showed that patients who received Tc-99m sestamibi or thallium-201 injections for MPI had evidence of DNA damage, compared to controls; however, repeated evaluations in the same patients (before/after radiotracer injection) were not available, and therefore a clear inference on the effect of ionizing radiation cannot be made. In the study by Varol et al. [10], among 27 children who underwent Tc-99m DMSA scintigraphy, DNA damage increased after the test, returning to normal levels after a week. Rief et al. [27] showed, by immunofluorescence, that strand breaks appeared after Tc-99m sestamibi injection for MPI and disappeared after 24 h. In the current study, a decrease of DNA damage with time could not be demonstrated, as patients were not re-evaluated later.

Additionally, even though DNA damage may occur, there are counteractive, self-protective mechanisms that contribute to reduce radiation effects. In fact, in response to DNA damage, cells activate repair genes [15]. Cheng et al. [28] have demonstrated that, after exposure to different types of ionizing radiation, the lymphocyte expression of mRNA of several repair genes was increased compared to controls. Won et al. [29] observed the activation of DNA repair pathways in patients who underwent MPI, by evaluating the phosphorylation of histone 2AX, protein p53 or serine/threonine protein kinase (ATM) in peripheral blood T lymphocytes by flow cytometry and immunohistochemistry, as well as the mRNA expression of repair genes such as BCL2 associated X, damage specific DNA binding protein 2, or Tp53 (a tumor-suppressing gene). Therefore, the biological consequences of DNA damage may be reduced by these mechanisms, helping minimize concerns about the effects of ionizing radiation used in MPI. Finally, new imaging protocols, using stress-only strategies, or new imaging hardware and software, which allow the use of very small radiotracer doses, may lead to further reductions in radiation-induced DNA damage from MPI.

### Limitations

As the collection of the second blood sample was relatively “early” regarding the half-life of Tc-99m sestamibi, the extent of DNA damage induced by the tracer might have been underestimated. This timing was due to the presence of the patients in the Nuclear Medicine laboratory, which typically lasts for up to 90 min. Nonetheless, as Rief et al. have described after performing multiple blood sample analyses, strand breaks can be detected as early as 5 min after radiotracer injection, without major difference when compared to the 1-h sample [27]. Therefore, we believe that our data may be representative of near-maximal radiation effects on the DNA. Additionally, as pointed by Azqueta et al. [30], DNA repair mechanisms can also occur very quickly, and experiments assessing DNA damage should take care to avoid repair of strand breaks; so, in this context, a shorter timing may also be desirable. Furthermore, due to the cross-sectional nature of this study, a return of damage levels to baseline could not be assessed, as other blood samples were not collected later. Finally, the study was performed with relatively high tracer doses, and therefore the amount of DNA damage might have been overestimated and may currently be substantially less with hardware improvement and new test protocols.

## Conclusions

In this patient sample, DNA damage was demonstrated in lymphocytes from patients undergoing MPI. However, most cells remained in class 0 damage after radiotracer injection. Further studies, including the evaluation of DNA repair, may help elucidate the genotoxic effects of MPI.

## Data Availability

The datasets generated and/or analyzed during the current study are not publicly available, as they have not been anonymized. However, they may be made available from the corresponding author on reasonable request.

## Abbreviations

ATM: Serine/threonine protein kinase
DI: Damage index
DMSA: Dimercaptosuccinic acid
EDTA: Ethylenediaminetetraacetic acid
MPI: Myocardial perfusion imaging
NCRP: National Council on Radiation Protection and Measurements
